# DANMEL: A manually curated reference database for analyzing mobile genetic elements associated with bacterial drug resistance

**DOI:** 10.1002/mlf2.12046

**Published:** 2022-12-11

**Authors:** Peng Wang, Xiaoyuan Jiang, Kai Mu, Ying Jing, Zhe Yin, Yujun Cui, Cuidan Li, Xinhua Luo, Fangzhou Chen, Ting Yu, Zhichen Zhu, Yansong Sun, Fei Chen, Dongsheng Zhou

**Affiliations:** ^1^ State Key Laboratory of Pathogen and Biosecurity Beijing Institute of Microbiology and Epidemiology Beijing China; ^2^ Beijing Institute of Genomics, Chinese Academy of Sciences China National Center for Bioinformation Beijing China; ^3^ University of Chinese Academy of Sciences Beijing China

## Abstract

We have developed a manually curated online reference database, DANMEL (http://124.239.252.254/danmel/), that addresses the lack of accurate dissection and annotation of the genetic structures of mobile genetic elements (MGEs) with genes for drug resistance. DANMEL contains accurately annotated and genetically dissected reference MGEs covering 5 categories and 135 subcategories/subfamilies of MGEs. Further, DANMEL provides a detailed guide on how to precisely annotate MGEs. DANMEL also provides SEARCH/BLAST functions to facilitate finding reference MGEs. Overall, DANMEL will aid researchers to conduct in‐depth genetic analysis of sequenced bacterial MGEs with drug‐resistance genes and further facilitate a better understanding of bacterial MGEs associated with drug resistance at a genomic level.

Accumulation and spread of genes with drug resistance among bacteria can lead to serious bacterial infections with substantially increased morbidity, mortality, and medical expenses, creating a significant public health concern globally^1^. Mobile genetic elements (MGEs), such as integrons, transposons, and plasmids, play a major role in the formation and spread of drug‐resistant bacteria through the exchange of drug‐resistance genes among bacteria^2^.

Currently, available MGE databases focus mostly on the collection and classification of MGEs with bacterial drug‐resistance genes (Table 1). INTEGRALL^3^, ISfinder^4^, and ICEberg^5^ are dedicated to the collection and classification of a large number of integrons, insertion elements (ISs), and integrative and conjugative elements (ICEs), respectively. ImmeDB^6^, ACLAME^7^, and VRprofile2^8^ all focus on collecting diverse categories of MGEs. Repositories of antibiotic resistance cassettes (RACs)^9^ and multiantibiotic resistance annotators (MARAs)^10^ collect transposons and provide transposon annotation service. TnCentral^11^ annotates transposons with gene organization (gene map), recombination sites, and open reading frames (ORFs). Although these databases collect and classify many MGEs, few of them provide detailed annotations. It is thus difficult to identify drug‐resistance‐related MGEs with insertion, deletion, truncation, or reversion using the existing databases. However, these special MGEs remain functional and thus the missing annotations for them may lead to a loss of critical information on the genetic structure.

**Table 1:** Comparison of DANMEL with existing databases.

Feature	INTEGRALL	ISfinder	ImmeDB	ACLAME	ICEberg	RAC	MARA	VRprofile2	TnCentral	DANMEL
MGE categories	Integron	IS	Prophage, transposon ICE, IME	Phage, plasmid	ICE	Transposon	Transposon	Transposon, ICE, plasmid	Transposon	Integron, transposon, ICE, IME, plasmid, etc.
High‐quality annotation	a	b	b	b	c	a	a	c	c	+
Gene organization	c	–	–	–	c	+	+	c	+	+
Download service	+	‐‐	+	+	c	+	+	+	c	+
MGE prediction	–	+	–	–	+	+	+	+	+	–

+, available; –, not applicable/available; a, only for gene cassette array; b, only for direct repeats (DRs) and inverted repeats (IRs); c, partially available; ICE, integrative and conjugative element; IME, integrative and mobilizable element; IS, Insertion element; MARA, multi‐antibiotic resistance annotator; RAC, repositories of antibiotic resistance cassette.

To accurately dissect and annotate the genetic structures of bacterial MGEs with drug‐resistance genes, we develop an online manually curated reference database called DANMEL. The database includes 377 representative reference MGEs with manually detailed annotations throughout the whole MGE sequence at a nucleotide level, which belong to five categories and 135 subcategories/subfamilies of MGEs. DANMEL also contains a guide for accurately annotating various types of bacterial MGEs that researchers can follow, using the high‐quality manually annotated sequences provided as reference, to precisely annotate their own MGEs. In addition, researchers can browse, search, and download all the entries in DANMEL. Overall, DANMEL is a powerful and helpful tool for researchers to explore bacterial MGEs with resistance genes at a genomic level.

## Scope and access of DANMEL

**Figure 1:**
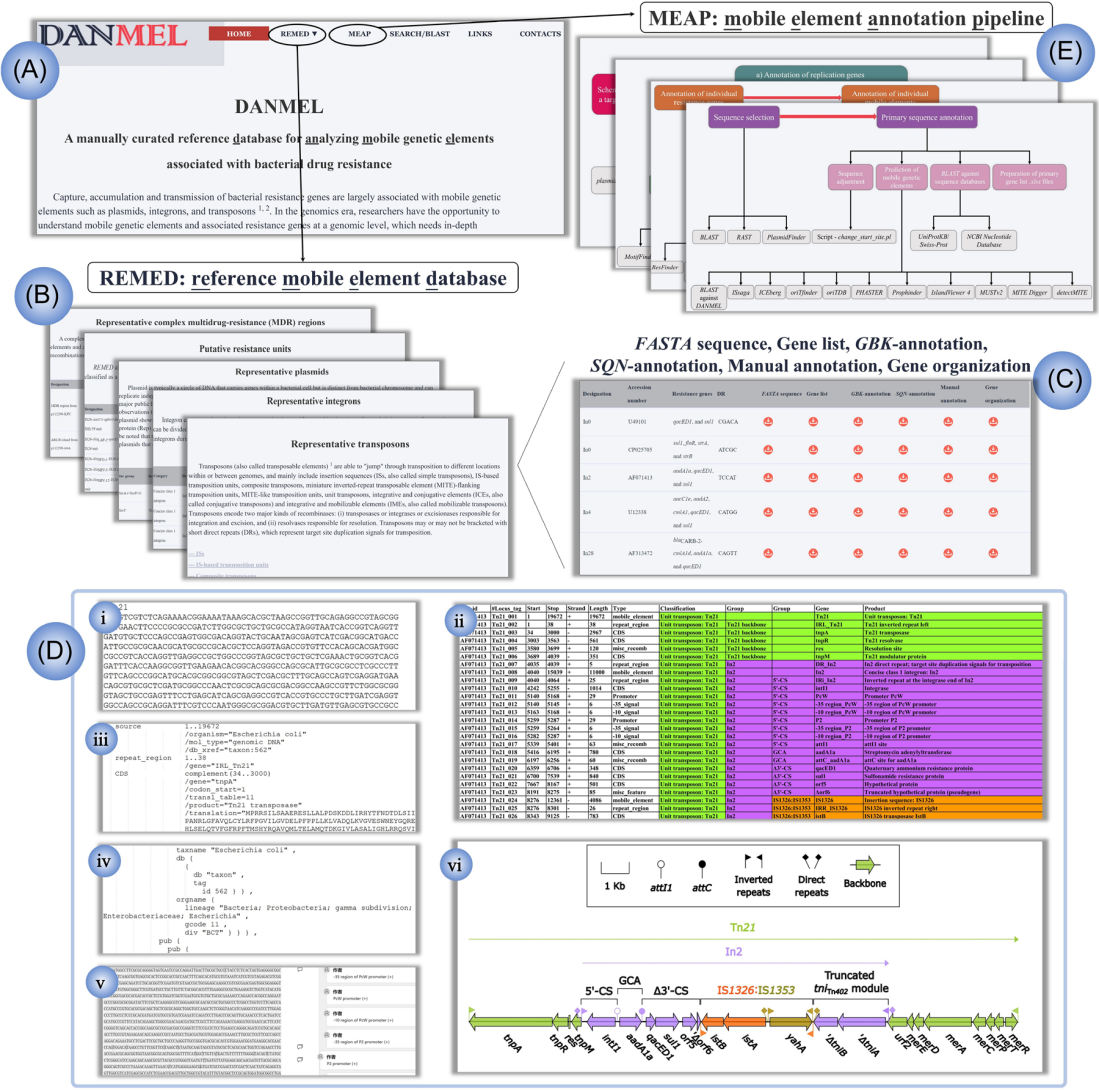
DANMEL interface. (A) DANMEL includes three major functional modules, REMED, MEAP, and SEARCH/BLAST, and menu items HOME, LINKS, and CONTACTS. (B) Five major categories of bacterial mobile genetic elements (MGEs), integrons, transposons, plasmids, resistance units, and complex multidrug resistance (MDR) regions, are included in REMED. (C)  Six types of reannotated files, FASTA sequence, gene list, GBK‐annotation, SQN‐annotation, manual annotation, and gene organization, are prepared for each MGE. All entries are available to be browsed and downloaded. Five‐step manual annotation in MEAP is composed of sequence collection and primary annotation; annotation of accessory modules; annotation of backbone sequences; drawing of gene organization diagrams; and data submission. (D) Tn*21* is taken as an example to present the six types of re‐annotated files (i–vi). (E) The five‐step manual annotation in MEAP is composed of sequence collection and primary annotation; annotation of accessory modules; annotation of backbone sequences; drawing of gene organization diagrams; and data submission.

DANMEL is constructed on a publicly available website (http://124.239.252.254/danmel/) and includes the major functional modules REMED (Reference Mobile Element Database), MEAP (Mobile Element Annotation Pipeline), and SEARCH/BLAST (Figure 1A). REMED contains 377 genetically dissected and manually annotated MGEs selected from PubMed and NCBI, including (i) representative MGEs that are widely used as references; (ii) prototype MGEs with complete genetic structures (less insertion, deletion, and/or reversion); (iii) MGEs with novel genetic structures; (iv) typical MGEs that play an important role in the dissemination of certain drug‐resistance genes; and (v) MGEs first identified/designated in our lab (more details in the Supporting Information), most of which are reference or prototype MGEs. MEAP is a guide for accurately annotating various types of bacterial MGEs, including 42 software/tools/databases^12^, ^13^, ^14^, ^15^ and in‐house scripts (Table S1). The SEARCH/BLAST model supports multiple search functions.

## Comprehensive representative bacterial MGEs in REMED

REMED includes 377 representative bacterial MGEs in five major MGE categories (Figure 1B): integrons (*n* = 45); transposons (*n* = 228), which include IS‐based transposition units (ISs), composite transposons, ICEs, and integrative and mobilizable elements (IMEs); plasmids (*n* = 53); putative resistance units (*n* = 44); and complex multidrug‐resistance (MDR) regions (*n* = 7). These five categories are further classified into 135 subcategories/subfamilies (Figure S1), covering a large proportion of known MGE types.

### Representative integrons

REMED contains 45 representative integrons according to different integrase (*intI*) genes, divided into class 1 (*n* = 38), class 2 (*n* = 5), and class 3 (*n* = 2) integrons (Figure S2). Class 1 integrons can be further divided into concise (*n* = 26) and complex (*n* = 12) class 1 integrons, the evolution and properties of which are shown in schematic maps.

#### Representative transposons

REMED covers 228 representative transposons belonging to eight major categories (79 subcategories): IS elements (*n* = 59); IS‐based transposition units (*n* = 16); composite transposons (*n* = 43); miniature inverted‐repeat transposable element (MITE) flanking transposition units (*n* = 1); MITE‐like transposition units (*n* = 1); unit transposons (*n* = 68); ICEs (*n* = 15); and IMEs (*n* = 25) (Figure S3).

##### Representative plasmids

REMED contains 53 representative plasmids belonging to 25 incompatibility (Inc) groups that correspond to all characterized Inc groups with completely determined sequences in *Enterobacteriaceae* and *Pseudomonas* ^16^, ^17^, ^18^. These include the earliest‐identified four Inc groups of plasmids (IncFII_pKF727591_, IncFII_pKp_Goe_414‐4_, Inc_pKPHS1_, and Inc_pRBL16_). Here, the modular structure of each plasmid typically consists of a backbone with one or more accessory modules at different sites in the backbone (Figure S4). REMED also lists key conserved backbone genes for replication, maintenance, and conjugal transfer in plasmids, and their nomenclatures are applied to other types of MGEs.

###### Putative resistance units

REMED contains 44 representative putative resistance units located in bacterial chromosomes and/or plasmids (Figure S5). Typical putative resistance units that have an IS element on one side (such as IS*CR1* and IS*CR2*) or two separate IS elements on both sides (such as IS*26* and IS*440*) are without flanking direct repeats (DRs) and cannot be classified as transposons, integrons, or plasmids. They can jump both within and among bacteria, taking adjacent regions and antibiotic resistance genes with them, as a whole element.

####### Representative complex MDR regions

REMED contains seven representative complex MDR regions assembled by sophisticated transposition and homologous recombination. Each of these regions has a complex mosaic structure harboring MDR genes with intact or partial integrons and/or transposons and/or putative resistance units (Figure S6). Complex MDR regions typically lead to the accumulation of multiple resistance genes in bacteria.

## Accurate and detailed manual annotation in REMED

The representative/prototypical MGEs mentioned above are manually reannotated as high‐quality references presented in a standard format and then subjected to the Sequin software and manual cross‐validation. REMED provides six types of annotation‐related files, including “*FASTA* sequence,” “gene list,” “*GBK*‐annotation,” “*SQN*‐annotation,” “manual annotation,” and “gene organization” (Figure 1C,D). “*FASTA* sequence” is the standardized FASTA file for the complete DNA sequence of each MGE. The “gene list” is an annotation file that contains the detailed features of all genes and the dissected modular structures composed of genes. The “gene list” is manually generated, including more detail than that in existing databases, and sites of the genes are manually verified to guarantee correctness. Further, different genetic modular structures are highlighted with different colors to show the structural relationships of the modular structure. For “*GBK*‐annotation” and “*SQN*‐annotation,” the standard *.gbk* and *.sqn* files are generated by Sequin^19^ based on the “gene list” to guarantee no validation errors. To facilitate extraction by users of the sequences for the single genes or modular structures in MGEs, the “manual annotation” file is generated by comments on the reannotated features for the DNA sequence of MGEs through manual interpretation. In “gene organization,” schematic vector diagrams are generated based on the “gene list” to visualize the modular structure of MGEs using our in‐house scripts.

In REMED, all genetic elements, such as DRs, inverted repeats (IRs), promoters, *attI* and *attC* sites, and ORFs, are annotated and their exact boundaries (“start” and “stop” sites) are listed in the “gene list” file. In existing databases, such as TnCentral, the above basic elements (DRs, IRs, and promoters) are ignored (Table 1 and Figure S7a). Further, DANMEL provides open access to all annotation files for users, while ICEberg only allows users to download the sequence files, although it provides similar high‐quality annotation for some ICEs (Table 1 and Figure S7b).

## Data processing in MEAP

MEAP is used to guide the annotation of various types of bacterial MGEs (Figure 1E). All 377 MGEs are manually annotated based on this pipeline to guarantee accuracy and correctness. Please also see our previous publications for examples of MGE annotations^20^.

Researchers start by comparing their target DNA sequence(s) with one or more genetically related sequences from DANMEL. The target sequence is then subjected to primary annotation, including raw annotation data generation, sequence adjustment, MGE prediction, BLAST against various sequence databases, and generation of primary annotation files. The representative MGEs deposited in DANMEL can be used as a reference for refining the primary annotations of the users’ own MGEs.

The modular structure of a target sequence (e.g., plasmid) is often divided into backbone sequences and accessory modules (defined as acquired DNA regions associated and bordered by MGEs and inserted at different sites of the backbone). Dissection of an accessory module includes the main annotations of DRs/IRs, conserved genes or markers, inserted intact/partial MGEs, and captured resistance genes. Here, as well as using available software and databases, extensive manual interpretation and gene site validation are needed because some special genetic structures with an insertion, deletion, truncation, or reversion may not be detected by the existing databases and the gene site prediction requires verification.

Dissection of a backbone module includes the identification of conserved key genes/markers responsible for replication (e.g., replication genes and iterons), maintenance (e.g., partition, and toxin–antitoxin), and conjugal transfer (type IV secretion system), which is achieved based on ORF prediction, conserved domain prediction, and BLAST against reference sequence databases, including DANMEL.

MEAP provides our in‐house scripts for generating diverse schematic maps to visualize the genetic structures. MEAP also requires the standardized annotation files to be checked using Sequin and strictly recognized by BankIt.

## Manual annotation quality control for MGEs

The 377 representative MGEs in REMED were all reannotated according to the steps of MEAP in DANMEL. The loci and boundary of genes have been manually verified by CLC Genomics Workbench and BLAST. The truncated genes/components are annotated by comparing them to related or reference MGEs. Thus, the correctness and details of DANMEL are guaranteed and outperform the annotations in existing databases. In addition, each of the 377 representative MGEs in REMED was manually reannotated by at least three experienced researchers, and the final annotation results were cross‐validated among these different researchers to minimize the chance of uncaught human errors.

## Multiple functional searches

On the SEARCH/BLAST page, a text‐based field option allows users to select between designation‐based searching and BLAST‐based searching. In designation‐based searching, users can input designations or accession numbers to search MGEs in DANMEL. The matching MGE and its associated information are presented in the results field. BLAST‐based searching is implemented using ViroBLAST. Specifically, the BLASTP option searches against the amino acid sequences of deposited key conserved plasmid backbone genes, while BLASTN searches against the nucleic acid sequences of all deposited MGEs. Users input the query sequence to search against all deposited nucleic acid sequences in DANMEL. The results field lists the matching MGEs with their identities and scores. Users can refer to these identities and scores to easily select the most appropriate MGE as the reference for annotating the queried sequences.

In summary, DANMEL is a specialized reference database for genetically dissecting and accurately annotating bacterial MGEs with drug‐resistance genes at a nucleotide level. Through the annotation guide in MEAP and using the entries in REMED as references, researchers should be able to precisely annotate their sequenced MGEs. Nonetheless, DANMEL is still a work in progress. In the future, we plan to include more reference/prototype bacterial MGEs, especially for Gram‐positive bacteria. Future efforts will also focus on creating an automatic annotation function, providing a powerful tool for precise, rapid, and real‐time analysis of various types of MGE sequence data.

## AUTHOR CONTRIBUTIONS

Dongsheng Zhou and Fei Chen conceived the study. Peng Wang, Xiaoyuan Jiang, Kai Mu, Ying Jing, Zhe Yin, Yujun Cui, Cuidan Li, Xinhua Luo, Fangzhou Chen, Ting Yu, and Zhichen Zhu analyzed the data. Peng Wang and Kai Mu constructed the website. Peng Wang, Kai Mu, and Xiaoyuan Jiang wrote the original draft, and Dongsheng Zhou, Fei Chen, and Yansong Sun reviewed the manuscript.

## ETHICS STATEMENT

Not applicable.

## CONFLICT OF INTERESTS

The authors declare no conflict of interests.

## Supporting information

Supporting information.

## Data Availability

DANMEL is publicly available at the online website: http://124.239.252.254/danmel/.
