# Supplementary material for: DANMEL: A manually curated reference database for analyzing mobile genetic elements associated with bacterial drug resistance
Source: mLife. 2022 Dec 11;1(4):460–4. doi: 10.1002/mlf2.12046 (PMC10989931; doi:10.1002/mlf2.12046)
Supplement: Supplementary file 1 — Supporting information. [file MLF2-1-460-s001.docx]

**SUPPLEMENTARY DATA**

**Materials and methods**

**MGE collection principles in DANMEL**

All the bacterial MGEs deposited in REMED are manually selected from PubMed and NCBI based on their representativeness, and essentially most of them is the reference or prototype MGEs. Five basic principles are proposed to guide the collection of representative MGEs: i) representative MGEs that are widely used as references in related work should be included, such as Tn*1696* ([1](#_ENREF_1)); ii) prototype MGEs with complete genetic structures (less insertion, deletion, and reversion) should be included, such as Tn*6535* ([2](#_ENREF_2)); iii) novel MGEs with novel genetic structures should be included, such as In1414 ([3](#_ENREF_3)); iv) typical MGEs always carrying a certain drug-resistant gene that play an important role in the dissemination of a certain drug-resistant gene, should be included, such as Tn*125* ([4](#_ENREF_4)) always carrying *bla*_NDM-1_ and is responsible for cthe wide dissemination of *bla*_NDM-1_ among bacteria; and v) the MGEs first identified/designated in our lab, are selectively included to this study. Each representative MEG included in REMED/DANMEL meets at least one of the abovementioned principles.

**Construction of website**

The website of DANMEL is constructed on a Linux v3.10.0 platform with an Apache2 v2.4.6 web server. The web interfaces are developed using HTML, CSS, JavaScript and PHP languages together with the jQuery library. The maintenance and query of the back-end database of DANMEL is based on MariaDB v5.5.68. Except for the logs and results generated by BLAST search. All the data in DANMEL can be browsed and downloaded freely and will be updated routinely.


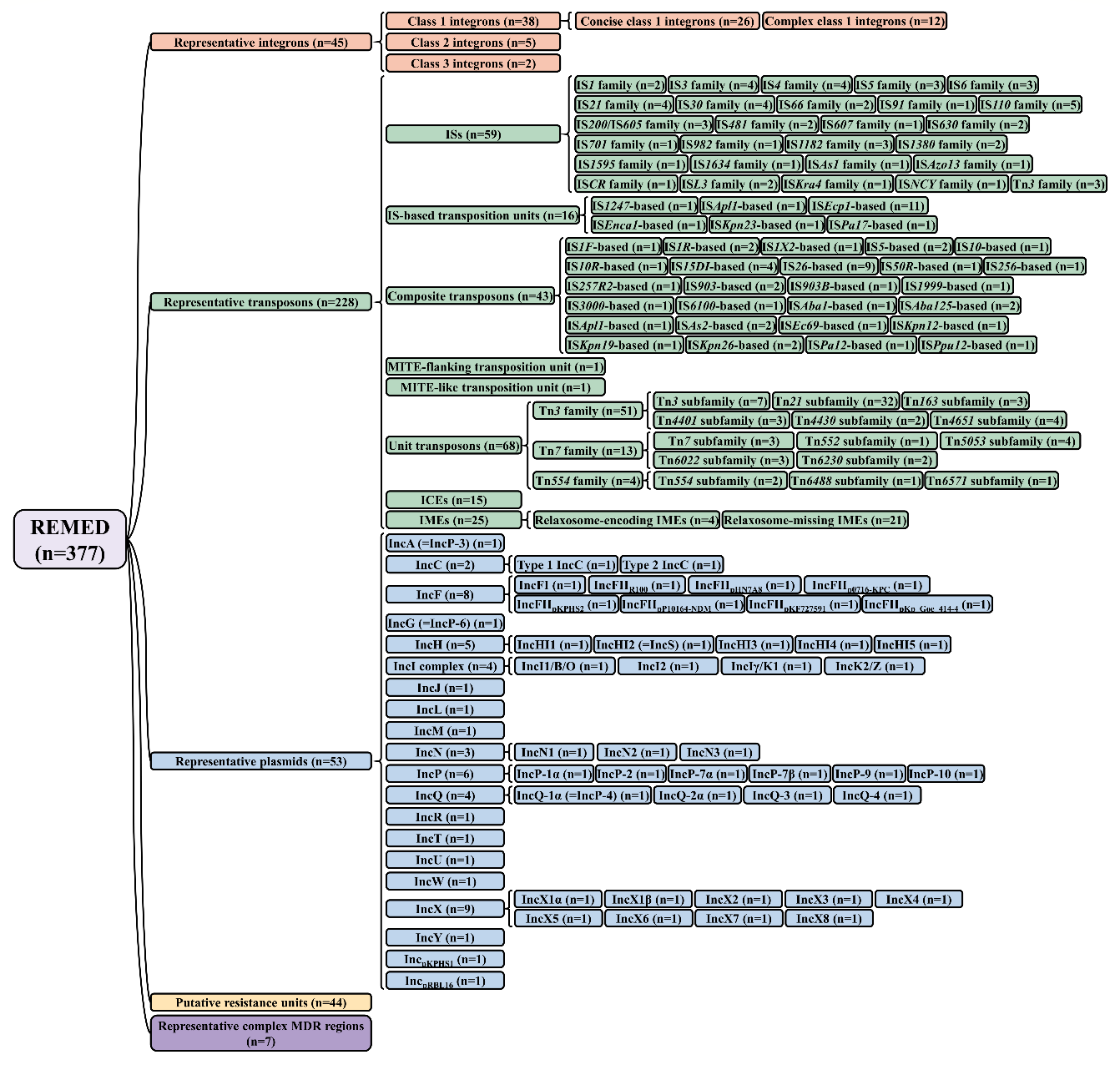


**Figure. S1. Classification of MGEs in REMED.** The 377 representative MGEs deposited in REMED can be assigned into the above-mentioned five major categories, and further divided into 134 sub-categories.


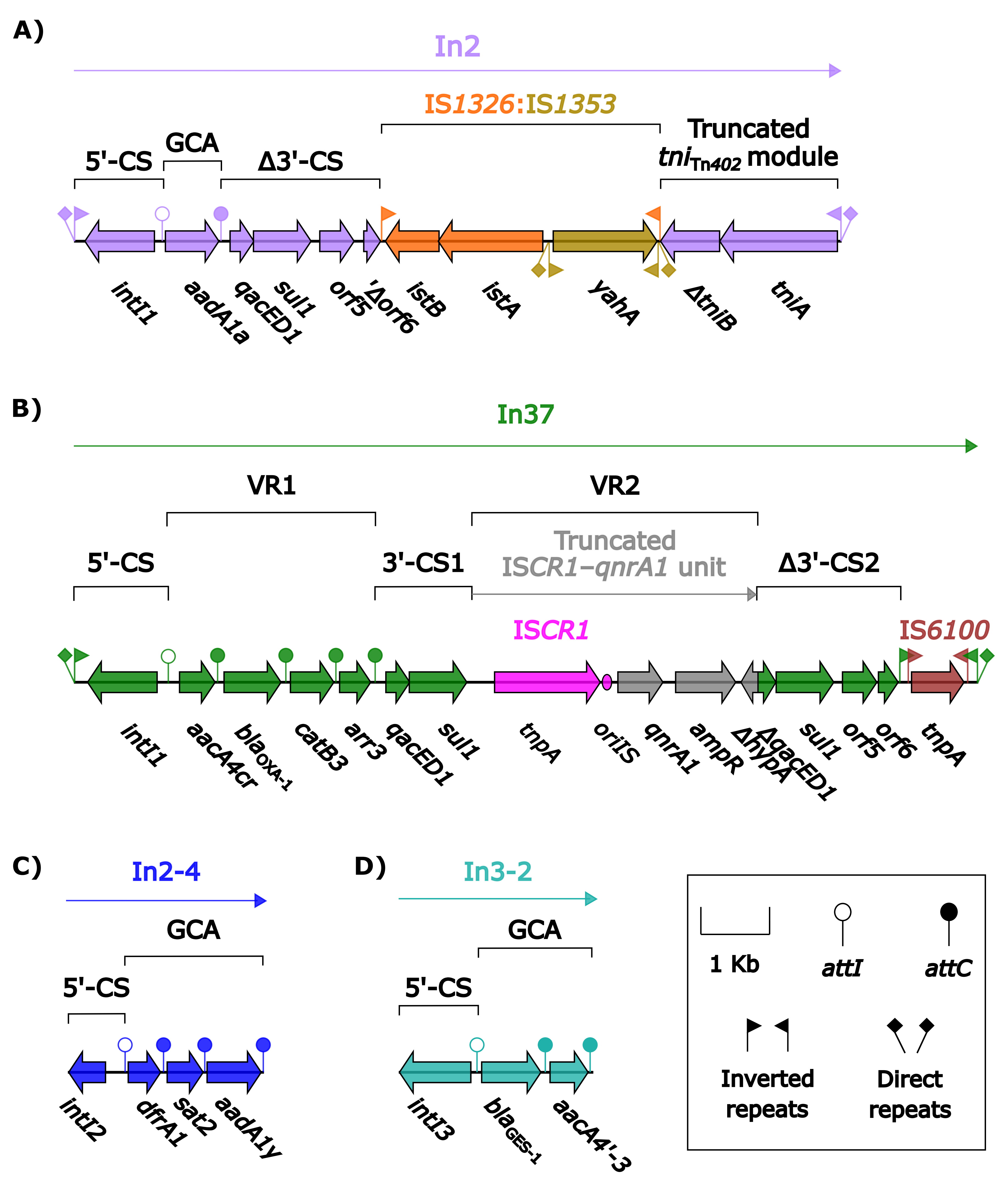


**Figure. S2. Gene organizations of four typical integrons.** Genes are denoted by arrows. Genes, mobile elements and other features are colored based on their functional classification. **A) In2 (AF071413):** a concise class 1 integron; **B) In37 (AY259086):** a complex class 1 integron; **C) In2-4 (AP002527):** a class 2 integron; **D) In3-2 (AY219651):** a class 3 integron.


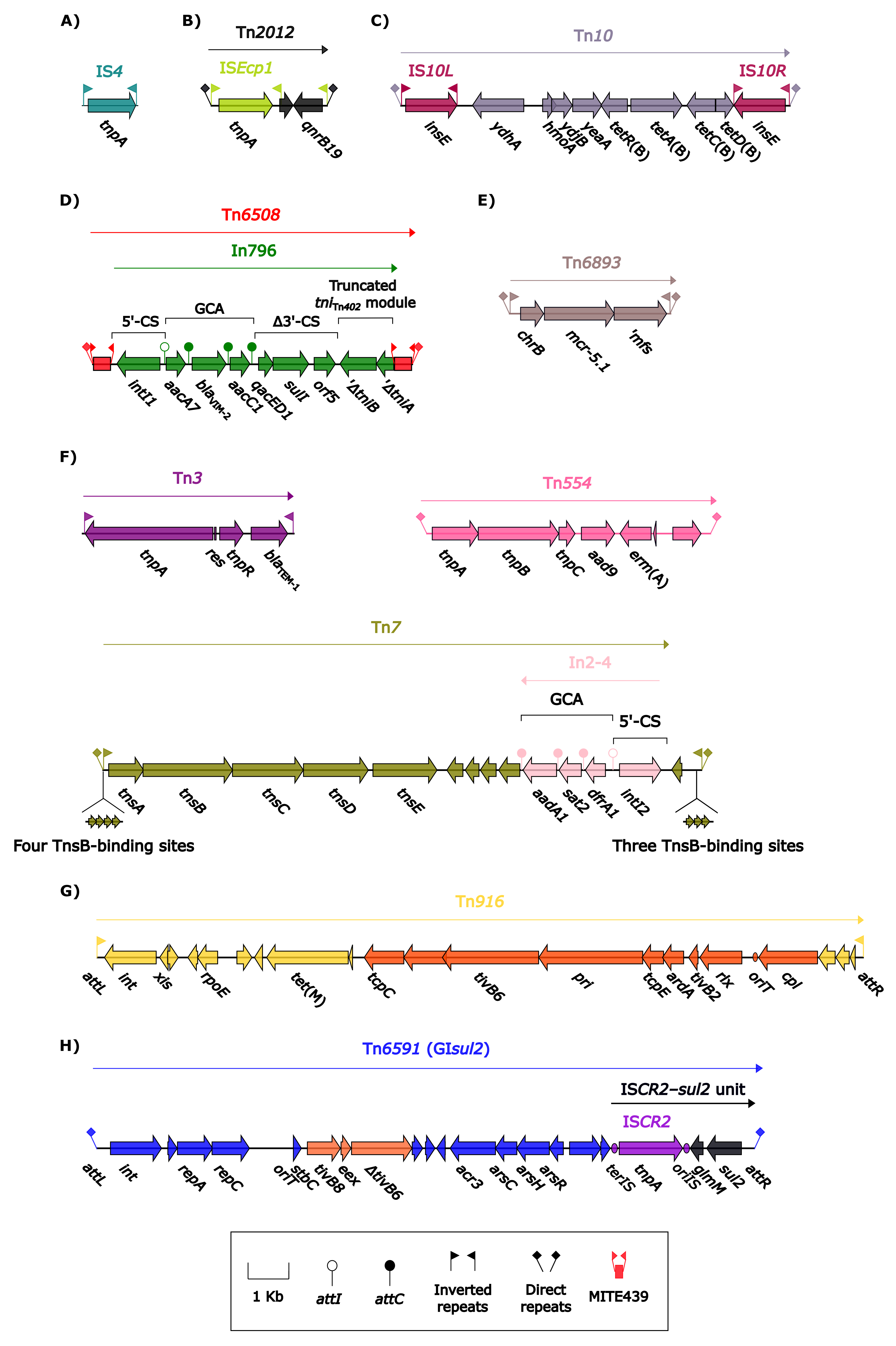


**Figure. S3. Gene organizations of 10 typical transposons.** Genes are denoted by arrows. Genes, mobile elements and other features are colored based on their functional classification. **A) IS*4* (J01733):** an insertion sequence (IS). ISs ([1](#_ENREF_1)) are small mobile genetic elements, each of which contains one or more transposase genes and is bordered by IRL (inverted repeat left) and IRR (inverted repeat right), and can be divided into 29 families; **B) Tn*2012* (EU523120):** an IS-based transposition unit. An IS-based transposition unit ([2](#_ENREF_2)) typically harbors an IS element together with the captured genes or genetic elements at its downstream end. Instead of the single IS element movement, the IS-based transposition unit is mobilized as one complete unit, which is bordered by IRL/IRR; **C) Tn*10* (AP000342):** a composite transposon. A composite transposon ([2](#_ENREF_2)) typically has a structure of an flanking accessory region by two separate IS elements, which may or may not be exact replicas and be typically bracketed by short DRs at both ends. Instead of IS element movement, the composite transposon is transposed as one complete unit; **D) Tn*6508* (JX235356):** a MITE-flanking transposition unit. MITEs ([3](#_ENREF_3)) are a group of non-autonomous transposable elements, and require other "helper" elements of transposases. MITEs have terminal IRL/IRR pairs and are flanked by short DRs. A MITE-flanking transposition unit typically has a structure of flanking an intact or incomplete genetic element (such as an integron ([4](#_ENREF_4))) by two copies of MITE, with short DRs bracketed at both ends. Instead of MITE movement, the MITE-flanking transposition unit transposes as one complete unit; **E) Tn*6893* (MH674200):** a MITE-like transposition unit. A MITE-like transposition unit is bracketed by IRL/IRR with short DRs at each extremity, and carries no transposase genes similar to MITEs ([5](#_ENREF_5)). In contrast to MITEs without accessory genes, this unit contains one or more accessory genes such as the antimicrobial resistance genes. This unit is trans-mobilized by recognizing its IRL/IRR by a transposase, displaying a mobilization mechanism similar to MITEs; **F) Tn*3* (HM749966), Tn*7* (KX117211), and Tn*554* (X03216):** unit transposons. A unit transposon carries a core transposition module, which can be mainly divided into Tn*3* ([6](#_ENREF_6)), Tn*7* ([7](#_ENREF_7), [8](#_ENREF_8))and Tn*554* ([9](#_ENREF_9)) families. Tn*3*-family core transposition module is composed of a transposase and a resolvase genes and a resolution (*res*) site; Tn*7*-family transposons exert transposition function through "cut-and-paste" mechanism, and encode five core transposition determinants, namely TnsA (endonuclease), TnsB (transposase), TnsC (transposition regulator), and TnsD plus TnsE (target-site selection proteins); Tn*554*-family transposon includes multiple transposase genes and is preferentially transposed to a single chromosomal site (e.g. *att554*), generating flanking DRs (e.g. GATGTA). Tn*3*- and Tn*7*-family transposons have terminal IRL/IRR pairs, but Tn*554* transposon carries no IRL/IRR pairs. The above three families can be further divided into 51, 13 and 4 subfamilies, respectively, based on the homology of transposase amino acid sequences; **G) Tn*916* (U09422):** an self-encoded integrative and conjugative element (ICE, also called a conjugative transposon). ICEs ([10](#_ENREF_10)) are primarily resided in the bacterial chromosome with the transferred ability between cells by conjugation. The core components of an ICE typically include *attL* (at the left end of the ICE), *int* [integrase: tyrosine or serine recombinase, or a DD(35)E transposase], *xis* (excisionase), *rlx*/*mob*, *nic*/*oriT* (nick site, origin of conjugative replication), *cpl* (TcpA- or VirD4-family coupling protein), a P (TivB)- or F (TivF)-type T4SS machinery (mating pair formation), and *attR* (at the right end of the ICE). A complete gene set for conjugal transfer include *rlx*/*mob*, *nic*/*oriT*, *cpl*, and the P- or F-type T4SS genes. ICEs are autonomous in both integration and conjugation; **H) Tn*6591* (GI*sul2*, AE014073):** an integrative and mobilizable element (IME, also called a mobilizable transposon). IMEs ([11](#_ENREF_11)) are defined as mobile genetic elements for encoding their own excision and integration. Although they do not include the T4SS system and a coupling protein, they are able to hijack or subvert the mating apparatus of related or unrelated conjugative elements (such as an IncA or IncC plasmid, or an ICE). IMEs are autonomous in integration but nonautonomous in conjugation, which are divided into two major subcategories: relaxosome-encoding and relaxosome-missing. Relaxosome-encoding IMEs have *attL*, *int*, *rlx*/*mob*, *nic*/*oriT*, and *attR*; the relaxase targets an *oriT* site and the resulting relaxosome is recruited to the T4SS together with a coupling protein by a co-resident conjugative element. Relaxosome-missing IMEs have *attL*, *int*, *nic*/*oriT*, and *attR*, but contain no conjugal transfer genes; a co-resident conjugative element recognizes the *nic*/*oriT* site of the IME, sufficiently making its intercellular transfer and subsequent transposition.


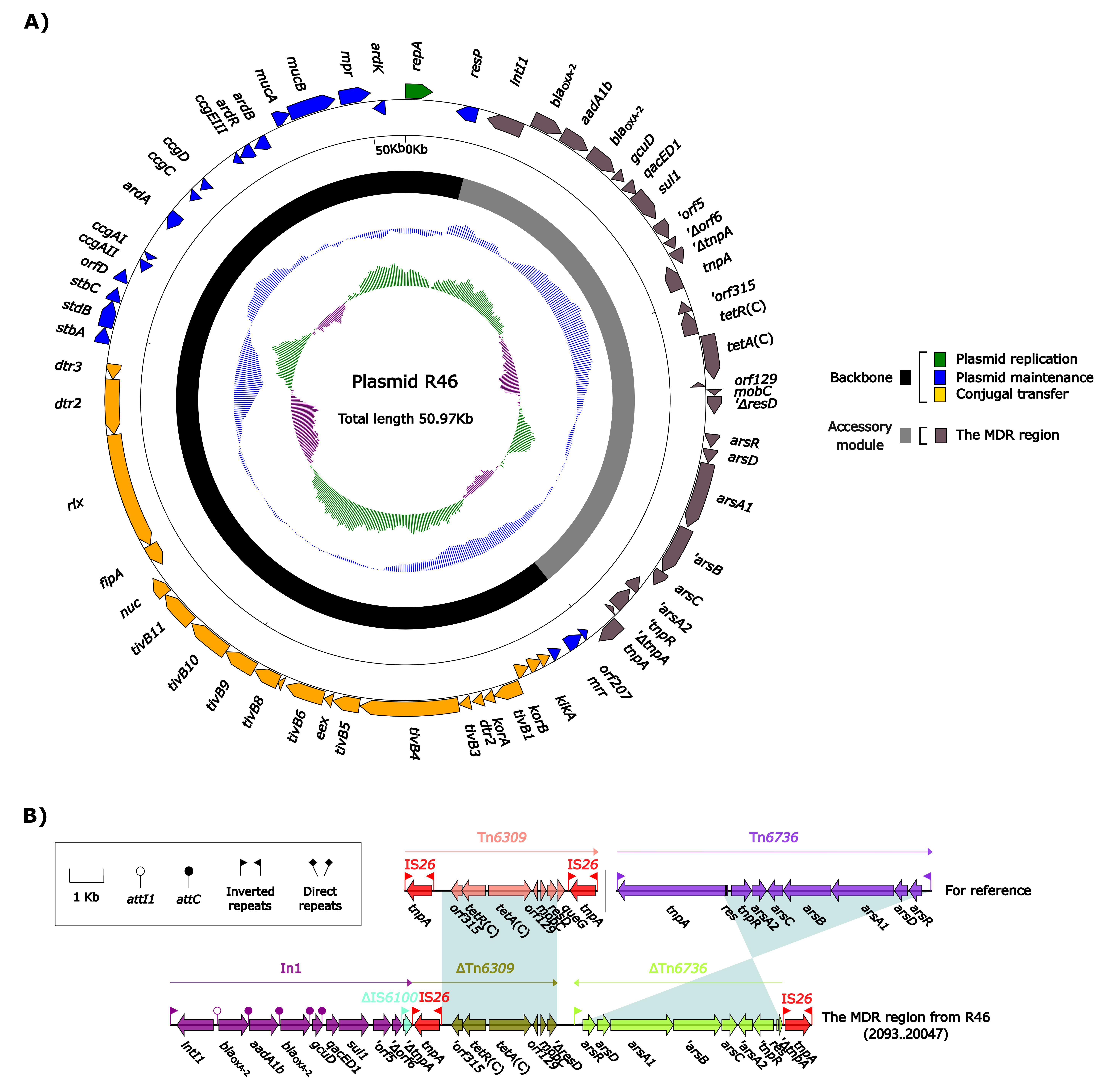


**Figure. S4. Modular structure of a typical plasmid R46. A) Schematic maps of plasmid R46 (AY046276).** Genes are denoted by arrows, which colors are determined based on gene function classification. The innermost circle presents GC-skew [(G-C)/(G+C)], with a window size of 500 bp and a step size of 20 bp. The next-to-innermost circle presents GC content. The modular structure of each plasmid is divided into the backbone as well as one or more separate accessory modules, which were defined as acquired DNA regions associated with mobile elements at different sites of the backbone. The backbone are further divided into the regions for plasmid replication (*repA* and its iterons), maintenance, and conjugal transfer ([12](#_ENREF_12), [13](#_ENREF_13)); **B)** **MDR region organization of R46.** Genes are denoted by arrows. Genes, mobile elements and other features are colored based on their functional classifications. Shading denotes regions of homology (nucleotide identity >95%).


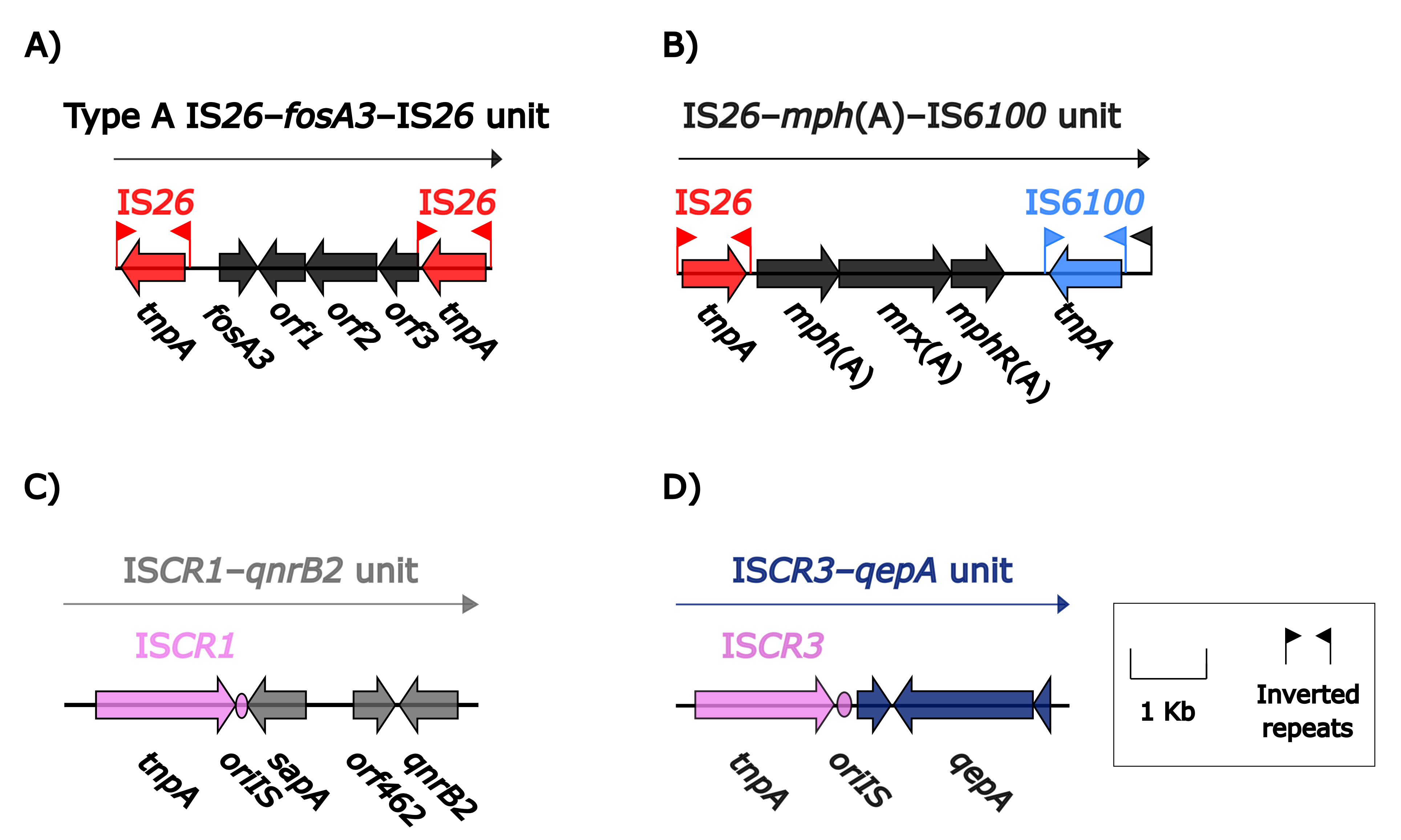


**Figure. S5. Gene organizations of four putative resistance units*.*** Genes are denoted by arrows. Genes, mobile elements and other features are colored based on their functional classification. **A) Type A IS*26*–*fosA3*–IS*26* unit (KP987215); B) IS*26*–*mph*(A)–IS*6100* unit (KY270851); C) IS*CR1*–*qnrB2* unit (EF219134); D) IS*CR3*–*qepA* unit (AB263754).**

**
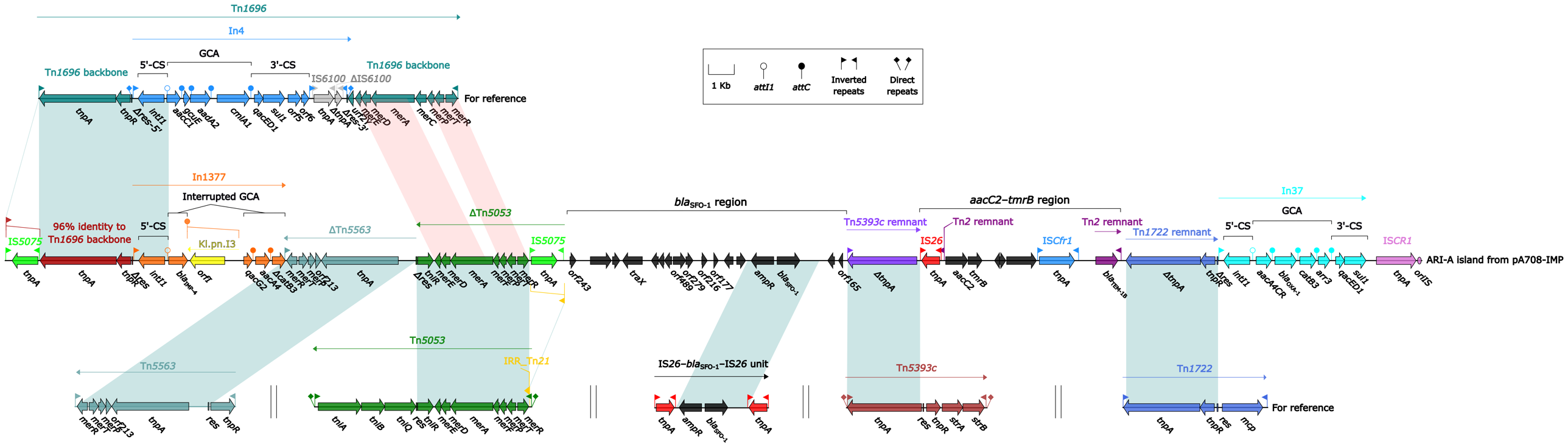
**

**Figure. S6. Modular structure of the MDR region from** **pA708-IMP.** Genes are denoted by arrows. Genes, mobile elements and other features are colored based on their functional classification. The MDR region from pA708-IMP (also designated as ARI-A island from pA708-IMP, MF344567) is composed of IRL (disrupted by IS*5075*)**–***tnpA***–***tnpR***–**Δ*res*_Tn_*_1696_*, In1377, ΔTn*5563*, ΔTn*5053*, *bla*_SFO-1_ region, Tn*5393c* remnant, *aacC2***–***tmrB* region, Tn*1722* remnant, and In37.

**
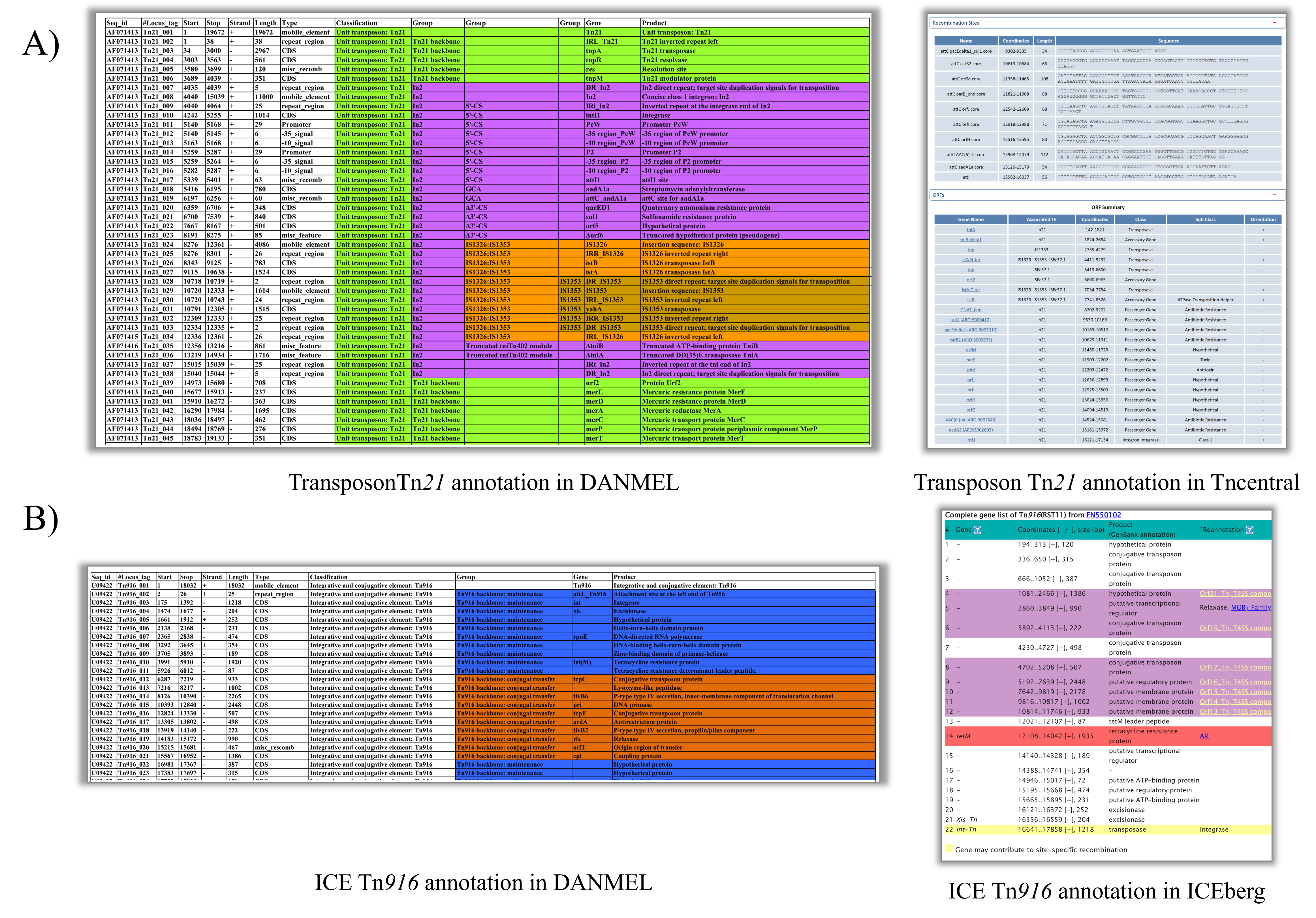
**

**Figure. S6. Annotation comparsion of DANMEL with Tncentral and ICEberg. A) an annotation comparsion of transposon Tn*21* annotation in DANMEL and in Tncentral. B) an annotation comparsion of ICE Tn*916* in DANMEL and in ICE berger**

**Table 1. Comparison of DANMEL with existing databases**

| Feature | INTEGRALL | ISfinder | ImmeDB | ACLAME | ICEberg | RAC | MARA | VRprofile2 | TnCentral | DANMEL |
| --- | --- | --- | --- | --- | --- | --- | --- | --- | --- | --- |
| MGE categories | Integron | IS | Prophage, Transposon ICE, IME | Phage, Plasmid | ICE | Transposon | Transposon | Transposon, ICE, plasmid | Transposon | Integron, Transposon, ICE, IME, Plasmid, etc. |
| high quality annotation | a | b | b | b | c | a | a | - | c | + |
| Gene organization | c | - | - | - | c | + | + | c | + | + |
| Download service | + | - | + | + | c | + | + | + | c | + |
| Annotation service | - | - | - | - | - | + | + | - | - | - |

^+^ Available, ^-^ Not applicable/available, ^a^ Only for gene cassette array; ^b^ Only for direct repeats and inverted repeats; ^c^ Partially available.

**Table S1. Softwars, tools, and in-house scripts collected in MEAP**

|  | Name | Description | Links |
| --- | --- | --- | --- |
| 1 | BLAST | BLAST finds regions of similarity between sequences. The program compares nucleotide or protein sequences to various sequence databases (such as NCBI Protein Database, Refseq_genomes, Refseq_protein, Swissprot) and calculates the statistical significance. | http://blast.ncbi.nlm.nih.gov/Blast.cgi |
| 2 | RAST | RAST is a fully automated service for annotating complete or nearly complete bacterial and archaeal genomes. It provides high quality genome annotations for these genomes across the whole phylogenetic tree and can identify genomic features (i.e., protein-encoding genes and RNA) and annotating their functions. | http://rast.nmpdr.org/ |
| 3 | PlasmidFinder | PlasmidFinder identifies the Inc group of a plasmid in total or partial sequenced isolates of bacteria. | https://cge.cbs.dtu.dk/services/PlasmidFinder/ |
| 4 | ISsaga | ISsaga is an ensemble of web-based methods for high throughput identification and semi-automatic annotation of insertion sequences in prokaryotic genomes. | http://issaga.biotoul.fr/ISsaga2/issaga_index.php |
| 5 | ICEberg | ICEberg is an integrated database that provides comprehensive information about integrative and ICEs found in bacteria. | http://db-mml.sjtu.edu.cn/ICEberg/ |
| 6 | oriTfinder | oriTfinder is a web-based tool that facilitates rapid identification of the origin site of DNA transfer (oriT) of a conjugative plasmid or a ICE or IME. | https://bioinfo-mml.sjtu.edu.cn/oriTfinder/ |
| 7 | oriTDB | oriTDB is a web-based database, which currently contains details of transfer origin regions (oriT), nicking sites (nic), relaxases and auxiliary DNA-binding proteins, type IV coupling proteins, as well as a collection of directly related references. | https://bioinfo-mml.sjtu.edu.cn/oriTDB/ |
| 8 | PHASTER | PHASTER is a web server for the rapid identification and annotation of prophage sequences within bacterial genomes and plasmids. | http://phaster.ca/ |
| 9 | Prophinder | Prophinder is a tool dedicated to the detection of prophages in sequenced bacterial genomes based on BLASTP against the ACLAME database (http://aclame.ulb.ac.be/). | http://aclame.ulb.ac.be/Tools/Prophinder/ |
| 10 | IslandViewer 4 | IslandViewer is a computational tool that integrates four different genomic island prediction methods: IslandPick, IslandPath-DIMOB, SIGI-HMM, and Islander. | http://www.pathogenomics.sfu.ca/islandviewer/browse/ |
| 11 | MUSTv2 | MUSTv2 is a program for de novo discovering miniature inverted-repeat transposable elements from genomic sequences. It is implemented in Perl/C++ program languages, and BioPerl library is also used to process the sequence files. | http://www.healthinformaticslab.org/supp/resources.php |
| 12 | MITE Digger | MITE Digger is a program for de novo discovering miniature inverted-repeat transposable elements from genomic sequences. It is implemented in Perl with Tk for graphic user interface for use on Microsoft Windows systems. | http://labs.csb.utoronto.ca/yang/MITEDigger/ |
| 13 | detectMITE | detectMITE is a tool based on MATLAB for detecting MITEs in genomes. | https://sourceforge.net/projects/detectmite/ |
| 14 | UniProtKB/Swiss-Prot | UniProtKB/Swiss-Prot is the manually annotated and reviewed section of the UniProt Knowledgebase (UniProtKB). It is a high quality annotated and non-redundant protein sequence database, which brings together experimental results, computed features and scientific conclusions. | http://web.expasy.org/docs/swiss-prot_guideline.html |
| 15 | NCBI Nucleotide Database | NCBI Nucleotide Database is a collection of nucleotide sequences from several sources, including GenBank, RefSeq, Third Party Annotation (TPA), and PDB. | https://www.ncbi.nlm.nih.gov/nucleotide/ |
| 16 | ResFinder | ResFinder is a web-based method, using BLAST for identification of acquired antimicrobial resistance genes and their resistance phenotypes in target sequences. | https://cge.cbs.dtu.dk/services/ResFinder/ |
| 17 | CARD | CARD is a bioinformatic database of resistance genes, their products and associated phenotypes. Perform standard BLAST searches against the CARD reference sequences. Results are annotated with extra information from CARD. | https://card.mcmaster.ca/analyze/blast |
| 18 | INTEGRALL | The INTEGRALL database is a freely available tool developed in order to provide an easy access to integron's DNA sequences and genetic arrangements. The INTEGRALL database only contains resistance genes carried by integrons. | http://integrall.bio.ua.pt/?search |
| 19 | RGI | RGI in CARD provides a preliminary annotation of DNA or protein sequence(s). | https://card.mcmaster.ca/analyze/rgi |
| 20 | RefSeqGene | RefSeqGene, a subset of RefSeq project, defines genomic sequences to be used as reference standards for well-characterized genes. | https://blast.ncbi.nlm.nih.gov/Blast.cgi?PROGRAM=blastn&BLAST_PROGRAMS=megaBlast&PAGE_TYPE=BlastSearch&SHOW_DEFAULTS=on&BLAST_SPEC=RefseqGene |
| 21 | BacMet | BacMet provides a high quality, manually curated database of experimentally confirmed antibacterial biocide- and metal-resistance genes, and includes a database of predicted resistance genes. | http://bacmet.biomedicine.gu.se/ |
| 22 | ISFinder | ISFinder provides a list of insertion sequences isolated from bacteria and archae, and also contains the search and BLAST functions. | https://www-is.biotoul.fr/index.php |
| 23 | Transposon Registry | Transposon Registry aims to simplify transposon nomenclature for new bacterial and archaeal elements and provide a searchable repository for all transposons to aid future research. | http://transposon.lstmed.ac.uk/ |
| 24 | BPROM | BPROM is a bacterial sigma70 promoter recognition program with about 80% accuracy and specificity. It is best used in regions immediately upstream from ORF start for improved gene and operon prediction in bacteria. | http://linux1.softberry.com/berry.phtml?topic=bprom&group=programs&subgroup=gfindb |
| 25 | NNPP | NNPP is a method that finds eukaryotic and prokaryotic promoters in a DNA sequence. | http://www.fruitfly.org/seq_tools/promoter.html |
| 26 | FAIR | FAIR aims to finding all internal repeats in nucleotide sequence(s). | http://bioserver1.physics.iisc.ernet.in/cgi-bin/fair4/fair/indx.pl |
| 27 | TRF | TRF is a program to locate and display tandem repeats in nucleotide sequence(s). | http://tandem.bu.edu/trf/trf.html |
| 28 | MotifFinder | MotifFinder finds out sequence motifs in the query sequence against Motif Libraries such as PROSITE, Pfam and NCBI-CDD, and provides functional and genomic information of the found motifs using DBGET and LinkDB as the hyperlinked annotations. | http://www.genome.jp/tools/motif/ |
| 29 | NCBI CD-search | CD-Search searches the Conserved Domain Database (NCBI-CDD; https://www.ncbi.nlm.nih.gov/cdd/) with protein or nucleotide query sequences. It uses RPS-BLAST to quick scan a set of pre-calculated position-specific scoring matrices with a protein query. | https://www.ncbi.nlm.nih.gov/Structure/cdd/wrpsb.cgi |
| 30 | Batch CD-Search | Batch CD-Search serves as both a web application and a script interface for a conserved domain search on multiple protein sequences. | https://www.ncbi.nlm.nih.gov/Structure/bwrpsb/bwrpsb.cgi |
| 31 | ORF finder | ORF finder searches for ORFs in the target DNA sequence. | https://www.ncbi.nlm.nih.gov/orffinder/ |
| 32 | MUMmer | MUMmer is a tool for rapidly aligning entire genomes, whether in complete or draft form. | http://mummer.sourceforge.net/ |
| 33 | ClonalFrameML | ClonalFrameML is a software package that performs efficient inference of recombination in bacterial genomes. | https://github.com/xavierdidelot/ClonalFrameML |
| 34 | MEGA | MEGA is an integrated tool for conducting automatic and manual sequence alignment, inferring phylogenetic trees, mining web-based databases, estimating rates of molecular evolution, and testing evolutionary hypotheses. | https://www.megasoftware.net/ |
| 35 | RAxML | RAxML is a program for sequential and parallel Maximum Likelihood based inference of large phylogenetic trees. It can also be used for post-analyses of sets of phylogenetic trees, analyses of alignments and evolutionary placement of short reads. | https://cme.h-its.org/exelixis/web/software/raxml/index.html |
| 36 | Sequin | Sequin is a stand-alone software tool for submitting and updating sequences to the *GenBank*, *EMBL*, and *DDBJ* databases. | <https://www.ncbi.nlm.nih.gov/Sequin/index.html> |
| 37 | BankIt | BankIt is a web-based sequence tool for submitting sequences to *GenBank*. | <https://www.ncbi.nlm.nih.gov/WebSub/?tool=genbank> |
| 38 | Change_start_site.pl | Changing the start site of a sequence in a FASTA file | http://124.239.252.254/danmel/MEAP.html |
| 39 | genbank2tab.pl | Translating a GBK file to a gene list TAB file |  |
| 40 | plasmid_circular_plot.v3.pl | Plotting a circular diagram of target plasmids |  |
| 41 | locus_compare_plot.test.pl | Plotting a accessory module diagram of multiple sequences |  |
|  | locus_compare_plot.v2.pl |  |  |
| 42 | align_compare_plot.v3_pseudo.pl | Plotting a linear comparison diagram of multiple sequences |  |

**Reference**

1. Siguier P, Perochon J, Lestrade L, Mahillon J, Chandler M. ISfinder: the reference centre for bacterial insertion sequences. Nucleic acids research. 2006;34(Database issue):D32-6.

2. Partridge SR. Analysis of antibiotic resistance regions in Gram-negative bacteria. FEMS Microbiol Rev. 2011;35(5):820-55.

3. Delihas N. Impact of small repeat sequences on bacterial genome evolution. Genome biology and evolution. 2011;3:959-73.

4. Domingues S, Toleman MA, Nielsen KM, da Silva GJ. Identical miniature inverted repeat transposable elements flank class 1 integrons in clinical isolates of Acinetobacter spp. J Clin Microbiol. 2013;51(7):2382-4.

5. Kieffer N, Nordmann P, Millemann Y, Poirel L. Functional Characterization of a Miniature Inverted Transposable Element at the Origin of mcr-5 Gene Acquisition in Escherichia coli. Antimicrob Agents Chemother. 2019;63(7).

6. Nicolas E, Lambin M, Dandoy D, Galloy C, Nguyen N, Oger CA, et al. The Tn*3*-family of Replicative Transposons. Microbiol Spectr. 2015;3(4).

7. Peters JE. Targeted transposition with Tn*7* elements: safe sites, mobile plasmids, CRISPR/Cas and beyond. Mol Microbiol. 2019;112(6):1635-44.

8. Peters JE, Fricker AD, Kapili BJ, Petassi MT. Heteromeric transposase elements: generators of genomic islands across diverse bacteria. Mol Microbiol. 2014;93(6):1084-92.

9. Haroche J, Allignet J, El Solh N. Tn*5406*, a new staphylococcal transposon conferring resistance to streptogramin a and related compounds including dalfopristin. Antimicrob Agents Chemother. 2002;46(8):2337-43.

10. Delavat F, Miyazaki R, Carraro N, Pradervand N, van der Meer JR. The hidden life of integrative and conjugative elements. FEMS Microbiol Rev. 2017;41(4):512-37.

11. Guédon G, Libante V, Coluzzi C, Payot S, Leblond-Bourget N. The Obscure World of Integrative and Mobilizable Elements, Highly Widespread Elements that Pirate Bacterial Conjugative Systems. 2017;8(11).

12. Jiang X, Yin Z, Yin X, Fang H, Sun Q, Tong Y, et al. Sequencing of bla(IMP)-Carrying IncN2 Plasmids, and Comparative Genomics of IncN2 Plasmids Harboring Class 1 Integrons. Front Cell Infect Microbiol. 2017;7:102.

13. Feng J, Qiu Y, Yin Z, Chen W, Yang H, Yang W, et al. Coexistence of a novel KPC-2-encoding MDR plasmid and an NDM-1-encoding pNDM-HN380-like plasmid in a clinical isolate of *Citrobacter freundii*. J Antimicrob Chemother. 2015;70(11):2987-91.
